# Buserelin alleviates chloride transport defect in human cystic fibrosis nasal epithelial cells

**DOI:** 10.1371/journal.pone.0187774

**Published:** 2017-11-16

**Authors:** Marie-Laure Calvez, Nathalie Benz, Florentin Huguet, Aude Saint-Pierre, Elise Rouillé, Christelle Coraux, Claude Férec, Mathieu Kerbiriou, Pascal Trouvé

**Affiliations:** 1 Inserm, UMR1078 "Génétique, Génomique Fonctionnelle et Biotechnologies", Univ Brest, EFS, IBSAM, Brest, France; 2 Université de Bretagne Occidentale, Faculté de Médecine et des sciences de la santé, Brest, France; 3 Association G Saleun, Brest, France; 4 Inserm UMRS903, CHU Maison Blanche, Reims, France; 5 CHRU Brest, Hôpital Morvan, Laboratoire de Génétique Moléculaire, Brest, France; 6 Etablissement Français du Sang—Bretagne, Brest, France; Emory University School of Medicine, UNITED STATES

## Abstract

Cystic fibrosis (CF) is the most common autosomal recessive disease in Caucasians caused by mutations in the gene encoding the Cystic Fibrosis Transmembrane conductance Regulator (CFTR) chloride (Cl-) channel regulated by protein kinases, phosphatases, divalent cations and by protein-protein interactions. Among protein-protein interactions, we previously showed that Annexin A5 (AnxA5) binds to CFTR and is involved in the channel localization within membranes and in its Cl- channel function. The deletion of phenylalanine at position 508 (F508del) is the most common mutation in CF which leads to an altered protein (F508del-CFTR) folding with a nascent protein retained within the ER and is quickly degraded. We previously showed that AnxA5 binds to F508del-CFTR and that its increased expression due to a Gonadoliberin (GnRH) augments Cl- efflux in cells expressing F508del-CFTR. The aim of the present work was to use the GnRH analog buserelin which is already used in medicine. Human nasal epithelial cells from controls and CF patients (F508del/F508del) were treated with buserelin and we show here that the treatment alleviates Cl- channel defects in CF cells. Using proteomics we highlighted some proteins explaining this result. Finally, we propose that buserelin is a potential new pharmaceutical compound that can be used in CF and that bronchus can be targeted since we show here that they express GnRH-R.

## Introduction

Cystic fibrosis (CF) is the most common autosomal recessive disease in Caucasians. It is caused by mutations in the gene encoding the Cystic Fibrosis Transmembrane Conductance Regulator (CFTR) chloride (Cl^-^) channel [1, 2]. The Wild-type CFTR (Wt-CFTR, mature form ~170-kDa) belongs to the ATP-binding cassette (ABC) transporter superfamily. It is an ATP-gated channel [3] expressed in the apical membrane of epithelial cells where it helps to maintain normal electrolyte and fluid balance across the cell membrane. Its Cl^-^ channel function is regulated by protein kinases, phosphatases, divalent cations such as calcium and magnesium and by protein-protein interactions [4–16]. Among the protein-protein interactions involving CFTR, we previously showed that Annexin A5 (AnxA5) binds to CFTR via its nucleotide binding domain 1, and that it is involved in the channel localization within membranes [14]. We also showed that this interaction with AnxA5 is functionally relevant because it favors the global Cl^-^ efflux through CFTR [15].

There are currently 2009 described mutations in the CFTR gene (http://www.genet.sickkids.on.ca/cftr/app). Among them, the deletion of a phenylalanine at position 508 (F508del) is the most common, associated with 70% of CF alleles [17]. This deletion leads to an altered protein (F508del-CFTR) folding, the nascent protein being retained within the ER and being quickly degraded. Only a small amount of F508del-CFTR reaches the plasma membrane [9, 18–20] where it exhibits a reduced Cl^-^ channel activity due to an open probability which is 15 times lower than that of Wt-CFTR [4, 21]. Furthermore, the F508del-CFTR channel is subjected to a faster turnover from the cell surface than Wt-CFTR [22]. In order to alleviate F508del-CFTR defects, its pharmacology has greatly evolved those last 10 years. Indeed, a large number of small molecules increasing the amount of CFTR protein in the plasma membrane or its channel activity has been identified (for review: [23]). These molecules are called correctors and potentiators, respectively. Correctors are of main importance regarding F508del-CFTR because they are aimed to favor its triggering to membranes. Several reported CFTR correctors were found to rescue F508del-CFTR in cell lines. Nevertheless, only corr-4a was efficient in primary bronchial epithelial cells from CF (F508del/F508del) patients, by increasing Cl^-^ secretion to nearly 8% [24]. This shows the importance to validate new molecules in human cells despite a positive role in cell lines. Several other active compounds were also discovered, such as miglustat [25], glafenine [26], latonduine [27].

The Vertex pharmaceutical company developed two compounds: Lumacaftor (VX-809) and Ivacaftor (VX-770). Lumacaftor is a CFTR corrector which improves F508del-CFTR maturation and F508del-CFTR-mediated currents [28]. In human bronchial epithelial cells (HBE) from patients (F508del/F508del), it increases CFTR maturation and CFTR-mediated Isc by 4-fold. In a phase IIa trial (ClinicalTrials.gov number NCT00865904), lumacaftor reduced sweat Cl^-^ levels in adult patients (F508del/F508del) but induced no significant changes in lung function or patient-reported outcomes were observed [29]. This study suggested that a combination of lumacaftor with ivacaftor is necessary to obtain sufficient clinical effects in patients. Ivacaftor is a CFTR potentiator which was found to potentiate G551D-CFTR-mediated currents by increasing CFTR’s opening probability. In primary cultures (G551D/F508del) and in HBE from F508del/F508del patients, it increases CFTR-mediated transepithelial currents. In clinical studies (ClinicalTrials.gov number NCT00909532, NCT00909727 and NCT01521338), ivacaftor was shown to significantly improve the lung function, the body mass index (BMI) and the mucociliary clearance in G551D/G551D patients. In cultured HBE, the application of ivacaftor increased the lumacaftor-corrected F508del-CFTR-mediated transepithelial currents [28]. This result was the rational of combining correctors and potentiators for CF therapy [28]. In a phase 2 study (ClinicalTrials.gov number NCT01225211), it was shown that lumacaftor/ivacaftor combination improved lung function and reduced the sweat Cl^-^ levels, in patients having a F508del mutation. US FDA approved the drug combination (Orkambi™) for CF (F508del/F508del) patients. Nevertheless, this combination presents notable issues regarding its efficacy. Indeed, the clinical outcomes of Orkambi are poor, likely due to a possible lumacaftor-ivacaftor interaction. Several recent *in vitro* studies demonstrated that ivacaftor interfered with the effect of lumacaftor on Phe508del-CFTR channel function [30]. It is now suggested that clinical outcomes should be improved by using personalized combination. As a result, despite advances in CF therapy, the identification of new compounds could help to design new molecular combinations, to alleviate F508del-CFTR defects.

In previous studies, we showed that AnxA5 binds to F508del-CFTR and that an increased AnxA5 expression, in cells expressing F508del-CFTR, augments Cl^-^ efflux via the channel due to an increased localization in the membrane [14, 15]. Indeed, AnxA5 which is a membrane binding protein is likely only acting through a stabilization of F508del-CFTR in membranes. Because these results were obtained after transfection of the cDNA of AnxA5, we further looked for a compound which could be used in human. Finally, we showed that Gonadotropin-Releasing Hormone (GnRH) induces an AnxA5 overexpression and subsequently an increased Cl^-^ channel function in F508del-CFTR cell lines [31]. In order to give these results therapeutical developments, the aim of the present study was to use the GnRH analog buserelin, which is already used in medical practice, on CF cells. Human nasal epithelial cells (HNEC) from controls and from CF patients (F508del/F508del) were treated with buserelin and we show here that the treatment alleviates Cl^-^ channel defects in CF HNEC. Using proteomic tools, we searched for differentially expressed proteins before and after buserelin treatment. We highlighted some proteins which could explain our results upon the function of CFTR. Finally, we propose that buserelin is a potential new pharmaceutical compound that can be used in CF and that bronchus can be targeted since we showed that they express GnRH-R.

## Materials and methods

### Cell cultures

HNEC from non-smokers healthy control (n = 6) and CF patients (n = 4) were collected after brushing the middle turbinate using a sterile brush (endocervical brush, diam 5.5, laboratoire Gyneas, France). Brushes were placed in transport media RPMI 1640 with Hepes and Glutamax I (Gibco, USA) supplemented with 2% antibiotic/antimycotic solution (ATB/ATM, Sigma Aldrich, USA) and brought to the laboratory. Nasal cells were removed from the brush and centrifuged (5 min, 300g, room temperature). Samples were washed twice and centrifuged (5 min, 300g, room temperature). Cell pellets were suspended in a differentiation medium composed of Dubelcco’s modified Eagle’s medium (DMEM), F12 (from Gibco, USA) and Bronchial epithelial cell growth medium (BEGM, Lonza, Switzerland; 1:1). Differentiation medium was supplemented with hEGF, epinephrine, BPE, hydrocortisone, insulin, tri-thyronine and transferrin. 1% ATB/ATM, 0.1 μM retinoic acid and 1.5 μg/ml bovine serum albumin (Sigma Aldrich, France) were added. To improve cell adhesion, culture plates, transwell-supports and snapwell-supports (12 mm, 0.4 μm pore size, polyester, Corning Costar, Sigma Aldrich) were coated with collagen from human placenta (Type IV, Sigma Aldrich, France). For cell differentiation, samples were seeded on transwells or snapwells at a density of 1.10^5^ cells per membrane and cultured up to confluence. The media was removed from the upper compartment and cells were grown at the air liquid interface (ALI) for 40 days.

HBE were obtained after surgery. Tissues were transported to the laboratory in transport media plus ATB/ATM. The samples were rinsed in PBS 1X and cleaned by microdissection to eliminate the parenchyma. The bronchi fragments were frozen in isopentane by nitrogen vapour or split in pieces and placed in suspension in RPMI 1640 culture media containing Pronase E (0,5 mg/ml, Sigma Aldrich, USA) overnight at 4°C. The enzymatic digestion was stopped by several baths in DMEM/F12 with ATB/ATM and fetal bovine serum (10%). Cell pellets were recovered by centrifugation (300g, 5 min) and suspended in differentiation medium. In order to improve cell adhesion, cell supports were coated with collagen (Type IV, Sigma Aldrich). For cell differentiation, samples were seeded on transwell or snapwell at a density of 1.105 cells per membrane and cultured up to confluence. Cells were grown at the air liquid interface during 30 days.

Human cells were obtained under authorization of the local ethics committee (“Comité de Protection des Personnes”, Number: DC-2015-2381).

In some experiments, CFBE41o-/F508del cells were used. They were cultured as previously described [31].

### Immunocytochemistry

To study the differentiation of the human respiratory epithelium the following antibodies were used [32]: Muc-5Ac (1:100, anti-hMuc5Ac, clone CLH2, Novacastra, Germany), β-tubulin (1:1000, anti- βTubulin, clone KMX-1, MM France, France), ZO-1 (1:20, anti-ZO-1, immunogen 334–364, clone ZO1-1A12, Invitrogen, USA), CFTR (1:50, anti-CFTR antibody 24–1, Bio-techne, USA) and GnRH-R (1:100, FL-328, monoclonal antibody IgG, Santa Cruz). Secondary anti-mouse and anti-rabbit antibodies, directly coupled to Cy3 or Alexa Fluor 488 (IgG H+L, 1:400, Jackson ImmunoResearchLaboratories) were used. Negative controls were realized by substituting the primary antibody by PBS-BSA 3%.

Transwell supports were fixed with cold methanol (-20°C, 10 min), washed with PBS 1X and blocked with PBS-BSA 3% (1 hour, room temperature). Samples were stained with primary antibody against Muc-5Ac (1:100), β-tubulin (1:1000) and ZO-1 (1:20) overnight at 4°C and rinsed 3 times in PBS 1X. Membranes were further incubated with the goat anti-mouse secondary antibody conjugated with Cy3 (1:400,1 h at room temperature). After three washes, membranes were mounted in Vectashield plus DAPI (Vector laboratories Inc., USA). Red fluorescence was observed on an Axiostar plus microscope (Zeiss Oberkochen, Germany).

Bronchial section frozen were washed four times with PBS 1X before their permeabilization by 0.02% Triton X-100 in PBS for 5 min. After permeabilization, nonspecific sites were blocked by 3% BSA in PBS for 60 min at room temperature. Cells were then stained with primary antibody directed against epithelial cells markers cytokeratin 13 (CK13) and Muc5Ac, against CFTR and GnRH-R. Next, samples were incubated with anti-mouse and anti-rabbit secondary antibodies coupled with Cy3 or Alexa Fluor 488 (60 min, room temperature). After final washes, samples were mounted in VectaShield + DAPI and viewed with a confocal laser-scanning microscope (LSM 510, Axio Observer, Plan-Apochromat 63X/1.40 oil; Carl Zeiss GmbH, Germany).

### RNA extraction and RT-PCR

Reverse transcription polymerase chain reaction (RT-PCR) was performed for the detection of GnRH-Receptor (GnRH-R) mRNA expression, using the following primers: GnRH-R (319 bp) forward: 5’-GACCTTGTCTGGAAAGATCC-3’, and reverse 5’-CAGGCTGATCACCACCATCA-3’. RNAs were extracted using an RNeasy Plus mini kit (QIAGEN, Canada), according to the manufacturer’s instructions. The amount of RNA was determined with a nanophotometer (Implen GmbH, Germany) and 500 ng of total RNA were reverse-transcribed using Superscript II enzyme (Invitrogen, USA). PCR reactions were performed using Hotstart Taq polymerase kit (QIAGEN, France) with following parameters: enzyme activation at 95°C for 15 min, denaturation at 95°C for 30 seconds, annealing at 57°C for 30 seconds, extension at 72°C for 30 seconds and a final extension at 72°C for 10 min, for 35 cycles. The amplified DNA was electrophoresed in an agarose gel (2%) and stained with Ethidium bromide. For positive control, Poly A+ RNA from human pituitary gland (Clontech, Member of Takara Bio Inc., Japan) was used. Negative controls were performed by replacing cDNA by water.

### Protein extraction and Western Blot analysis

When confluent, cells were harvested after trypsin dissociation and lysed in NP40 cell lysis buffer (Invitrogen), composed of 50 mM Tris–HCl at pH 7.4, 250 mM NaCl, 5 mM, EDTA, 50 mM NaF, 1 mM Na3VO4, 1% Nonidet P-40 and containing complete protease inhibitor cocktail (Roche, Switzerland). The protein concentration was determined by Lowry’s methodology using BSA as a standard. Proteins were separated by SDS-PAGE (10%) and transferred onto PVDF membranes (GE Healthcare, UK). Blots were blocked for 1 hour in blocking buffer (5% non-fat dried skimmed milk in TBST (Tris-buffered saline plus 0.1% Tween20)). Membranes were incubated overnight at 4°C with mouse monoclonal antibody anti-GnRH-R (1:150, LH-RH Receptor Ab-3 clone GnRH03, MM-France, France) and with HRP-conjugated secondary antibodies (1:20000, Santa-Cruz Biotechnologie, UK). Bands were visualised by enhanced chemioluniscence (Ecl Plus, GE Healthcare).

### Patch-clamp analysis

Whole-cell patch clamp recordings of non-CF and CF HNEC, with and without Buserelin treatment, were performed using a Port-a-Patch system (Nanion Technologies GmbH, Germany) with an external amplifier unit HEKA EPC-10 [16, 31, 33]. All experiments were performed at room temperature. A voltage clamp protocol was carried out between -80 to +80 mV (10 mV steps) with a holding membrane potential of -80 mV. For recordings, the cell pellets were suspended in external solution (pH 7.4, 288 mOsm) contained the following (in mM): 140 NaCl, 2 CaCl_2_, 1 MgCl_2_, 10 Hepes, 5 D-glucose monohydrate. The internal solution (pH 7.2, 285 mOsm) contained the following (in mM): 50 CsCl, 10 NaCl, 60 Cs-Fluoride, 20 EGTA, 10 Hepes/CsOH and 5 Mg-ATP (Mg salt). CFTR activators (10 μM foskolin and 30 μM genistein, Sigma Aldrich) and inhibitor (5 μM CFTRinh172, Sigma Aldrich) were added to activate or inhibit CFTR, respectively.

### Ussing chamber

HNEC were grown at ALI for 40 days until complete differentiation. Confluent and resistive cells grown on snapwells were treated with apical buserelin at 10^-12^M for 1h, 2h, 4h and 8h. They were mounted in Ussing Chamber (WPI, UK), incubated at 37°C and gassed with 95% O_2_/5% CO_2_. For all measurements a Cl^-^ gradient was applied by different composition of luminal and basal Krebs solution. The luminal buffer contained (in mM): 107 K-gluconate, 4.5 KCl, 25 NaHCO_3_, 1 MgSO_4_, 1.8 Na_2_HPO_4_, 0.2 NaH_2_PO_4_, 5.75 Ca-gluconate, 12 D-glucose. The basal buffer contained (in mM, pH7.4): 111.5 KCl, 25 NaHCO_3_, 1 MgSO_4_, 1.8 Na_2_HPO4, 0.2 NaH_2_PO_4_, 1.25 CaCl_2_, 12 D-glucose. Voltage-sensing electrode consisted of 3M KCl-agar bridges. The short-circuit current (Isc) was monitored continuously using a DVC-1000 Voltage-Clamp (WPI, UK). The potential differences and the transepithelial resistances were measured at baseline conditions. After stabilisation of baseline current (10–20 minutes), pharmacologic agents (amiloride, forskolin, genistein, CFTRinh172) were added sequentially to the apical and/or basolateral side. Forskolin (10 μM) was added to the apical and basolateral bath, whereas amiloride (100 μM), genistein (50 μM) and CFTRinh172 (5 μM) to the apical bath. The effect of buserelin (10^−12^ M) was measured and data were collected and analysed with DataTrax software (WPI, UK).

### cAMP measurements

Transwell supports were incubated with or without Buserelin (10^−12^ M, 2h) and lysed in 0.1 M HCl. cAMP levels were measured by an Elisa kit (Enzo life sciences, Switzerland), according to the manufacturer’s instructions.

### Two-dimensional gel electrophoresis (2-DE) and mass spectrometry (MS)

Treated (Buserelin, 10^−12^ M, 2h) and untreated HNEC were rinsed twice with cold PBS 1X before being scrapped. Cells were lysed in 2-D extraction buffer-V supplemented with 40 mM DTT, 1:100 protease inhibitor mix, 2% IPG buffer (GE Healthcare, UK). Total protein concentrations were determined using a 2-D Quant-kit (GE Healthcare, UK). 100 μg of total proteins were used in each experiment. Protein samples were diluted in DeStreak Rehydration Solution (GE Healthcare, UK). Passive rehydration of Immobiline DryStrip gels (7 cm, pH 3–10 NL, GE Healthcare, UK) with samples was performed (8 hours). Immobiline DryStrip gels were transferred to an Ettan IPGphor 3 (GE Healthcare, UK) for first dimension isoelectric focusing (IEF). The used 5 steps IEF protocol was Step 300 V, 200 V/h; Grad 1000 V, 300 V/h; Grad 5000 V, 4500 V/h; Step 5000V, 9000 V/h; Step 300V. Second-dimension separation was performed immediately after IEF. Immobiline DryStrip gels were equilibrated (15 min at room temperature) in two SDS equilibration buffers (6 M urea, 75 mM Tris-HCl pH = 8.8, 30% Glycerol, 2% SDS, 0.0002% bromophenol blue plus DTT or iodoacetamide) before SDS-PAGE. Electrophoresis was performed in 1X Tris-Glycine-SDS buffer (BioRad, USA) at 120 V for 2.5 hours. The 2-DE gels were stained with a G-250 Coomassie blue solution, scanned and digitalized using a GS-800 Calibrated Densitometer (Bio-Rad, USA). Gel alignment, spots detection and quantification were done using the PDQuest software (PDQuest Basic-8.0.1, BioRad, USA). Data are the means of 4 gels from 4 different experiments.

MS analysis of the differentially expressed proteins was performed at the « Plateforme d’Analyse Protéomique de Paris Sud Ouest, France » facility, using LC-MS/MS. Enzymatic digestion was realised by 50ng Trypsin (enzyme/protein ratio: 1/50, 37°C, overnight). Supernatant was used for extraction. LC-MS/MS analysis was done using a LTQ-Orbitrap Spectrometer. Nano HPLC (Dionex RSLC UltiMate3000) was used, followed by MS (Scan range: 400–1500 m/z, fragmentation: CID, Energy of collision: 35%, Cycle for fragmentation (TopN): Top8). Data were converted to mzXML using MS convert (ProteoWizard v 3.0.8934). Databases interrogation was performed by X!Tandem Piledriver (v2015.04.01.1) and X!Tandem Pipeline (v 3.4.2 ≪ Elastine Durcie ≫).

### Statistics

Statistical analyses were performed using the R software. Difference in means between two groups was tested using a Student t-test. To assess the difference between several groups, we applied a non-parametric test due to the non-homogeneity of variances. We used a Kruskal-Wallis test to assess the difference in medians between experimental conditions. In the post-hoc analysis, we tested one-by-one comparison using the Dunn test. To control for multiple testing, the raw p-values obtained from this analysis were adjusted with the q-value method via a false discovery rate (FDR) approach. Adjusted significance levels were calculated using the Dunn test command in R. To test if the CF HNEC at the basal level varied significantly between untreated and each time point (1, 2 and 4h), the significance levels were adjusted for three comparisons (Untreated vs 1h; untreated vs 2h, untreated vs 4h). A similar approach was used to test the effect of treatment with Fsk/Gst and CFTRinh172 at various time points. Results are expressed as the means ±SEM of n observations. Differences were considered statistically significant when p<0.001 (***); p<0.01 (**) and p<0.05 (*).

## Results and discussion

**Fig 1:**
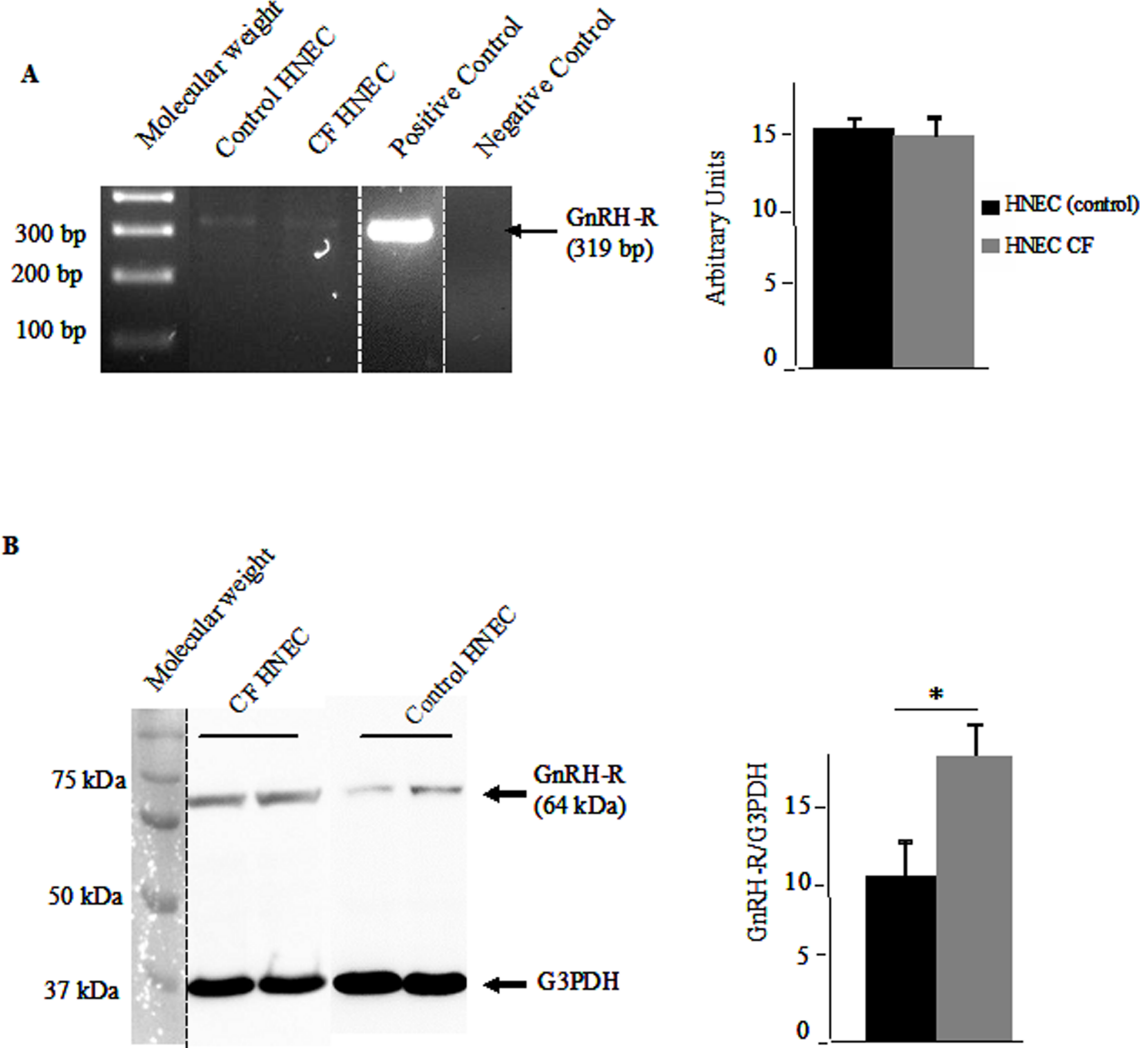
Basal mRNA and protein expression of GnRH-receptor (GnRH-R) in normal and CF HNEC. **A.** The left panel shows representative PCR bands for GnRH-R (319 bp) expression (2% agarose gel). A single band is observed in control HNEC, CF HNEC and human pituitary gland (positive control, spliced for the final figure). No signal is observed in the negative control. The bar graphs (right panel) represents the quantitative analysis of the mRNA expression (n = 4). No difference was observed between normal HNEC (expressing Wt-CFTR) and CF HNEC (expressing F508del-CFTR). **B**. The left panel shows a representative immunoblot analysis of the expression of GnRH-R (64 kDa) and G3PDH (37 kDa) proteins, in CF and control HNEC. Molecular weight was spliced for the final figure. G3PDH was detected to show that the loading was identical in each lane and to normalize the expression of GnRH-R. A quantitative analysis was performed (n = 4) and is represented as bar graphs (right panel). A significant increased expression of GnRH-R was observed in CF HNEC, when compared to normal HNEC.

Because we planned to use a GnRH analog (Buserelin) [34], we first had to show the expression of the GnRH-R in HNEC. The primary role of GnRH is the endocrine control of reproduction. Released from the hypothalamus, it acts upon the pituitary to stimulate LH and FSH secretion. Therefore, the presence of GnRH-R in extra-pituitary cells such as HNEC had to be assessed. First, we analyzed the mRNA expression of the receptor in normal and CF HNEC. Representative PCR bands for GnRH-R in control and CF HNEC are shown in Fig 1A (left panel). The quantification of the bands was performed and the result is presented as bar graphs (Fig 1A, right panel). No significant difference was observed between normal and CF HNEC. The expression of the GnRH-R protein was assessed by western blotting. As shown in Fig 1B (left panel), GnRH-R is expressed in normal HNEC and CF HNEC. A quantitative analysis was performed and a significant increased expression of GnRH-R was observed in CF HNEC, when compared to normal HNEC (Fig 1B, right panel). Our results are in accordance with previous results showing a GnRH-R expression in extrapituitary tissues [35]. Whereas high amounts of the mRNA were observed in prostate, thymus, and kidney [35], it was observed at lower levels in other organs such as heart, brain, placenta, lung, liver, skeletal muscle, pancreas, colon, ovary, small intestine, spleen, and testis [36]. In accordance with previous results showing the presence of GnRH-R in airway epithelial cells such as Calu-3 cells [37], 16HBE14 o^-^ and CFBE41o^-^ cells [31, 38], we observed the expression of GnRH-R protein in HNEC. Despite the observed expression in lung, the exact physiological role of GnRH-R in this tissue remains elusive.

**Fig 2:**
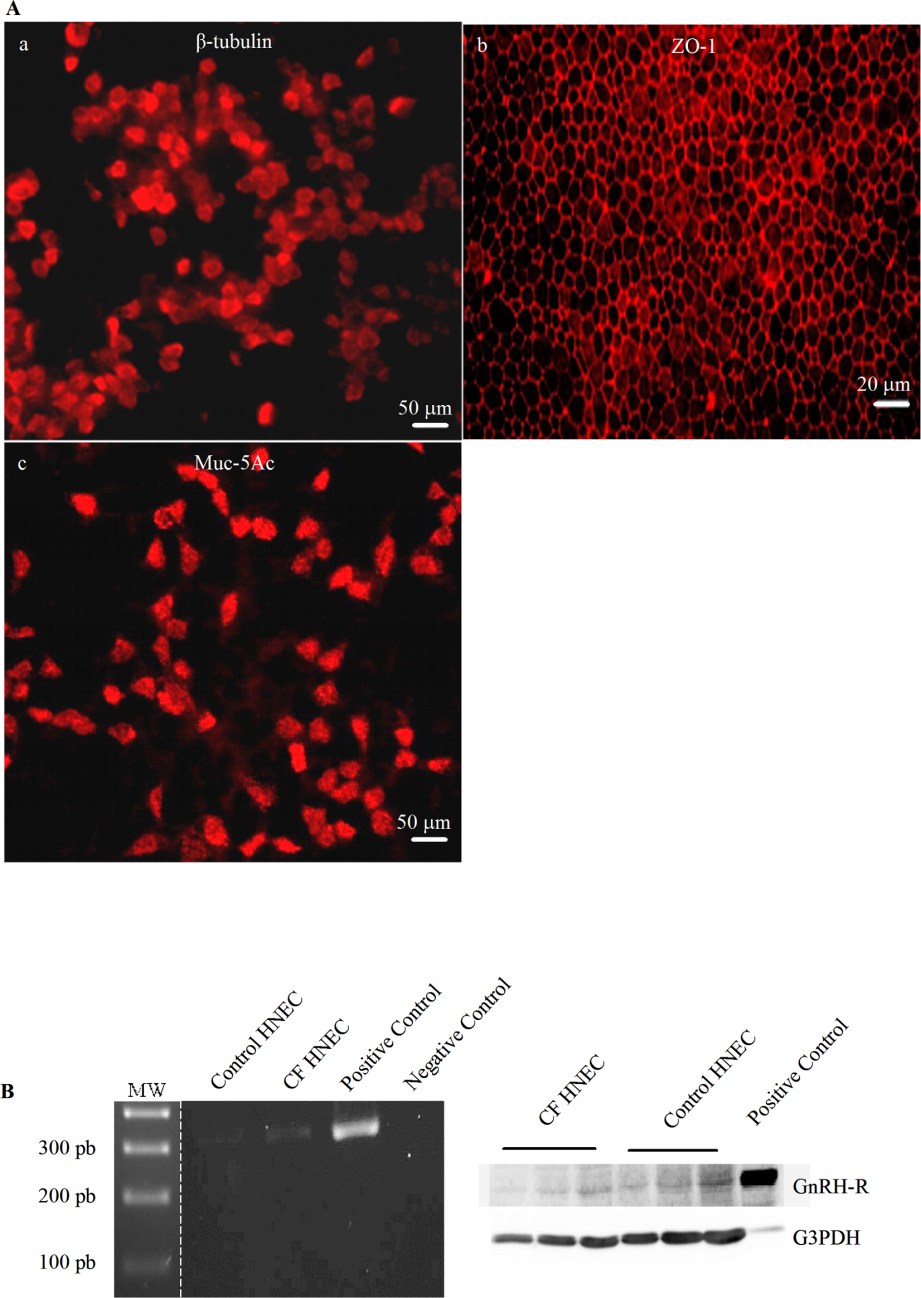
Characterization of the cell types and detection of GnRH-R in ALI cultures of HNEC. **A.** Cells were cultured at ALI for 40 days and were stained using antibodies directed against β-tubulin, ZO-1 and Muc-5Ac in order to label ciliated cells (a), tight junctions (b) and goblet cells (C), respectively. **B**. mRNA and protein expression of GnRHR were assessed in control and in CF HNEC in ALI cultures. The left panel shows representative PCR bands for GnRH-R (319 bp) expression in HNEC ALI cultures. A single band was observed in control HNEC, CF HNEC and human pituitary gland (positive control). No signal was observed in the negative control. The right panel shows representative immunoblots of GnRH-R (64 kDa) and G3PDH (37 kDa) protein expression, in ALI culture. The receptor was detected in CF HNEC, control HNEC and in CFBE41o-/F508del (positive control) cells. For each cell type, 40, 80 and 120 μg were loaded. G3PDH was detected as a loading control. Molecular weight was spliced for the final figure.

Non-CF and CF HNEC were cultured at ALI and the resulting cultures were characterized [39]. When they were covered by ciliated cells (*β*-tubulin-positive cells, ∼70%), they were considered differentiated. In order to characterize the cells after ALI cultures, ciliated cells, tight junctions and goblet cells were stained by immunohistochemistry, using antibodies directed against β-tubulin, ZO-1 and Muc-5Ac [33, 39]. An example of the labelling showing the reconstitution of the epithelium, performed in non-CF cells, is given in Fig 2A (a-c). In order to assess the GnRH-R expression in ALI cultures, its mRNA and protein expression of GnRH-R was assessed in control and CF HNEC. Representative PCR bands of GnRH-R (319 bp) in control and CF HNEC and in human pituitary gland (positive control) are shown in Fig 2B (left panel). A representative immunoblots showing the expression of the GnRH-R (64 kDa) and G3PDH (37 kDa) protein in control HNEC, CF HNEC and CFBE41o-/F508del cells (positive control) is shown in Fig 2B. We observed that ALI cultures do not modify GnRH-R expression and that our HNEC cultures in ALI were in accordance with previous results [33], validating our methodology.

**Fig 3:**
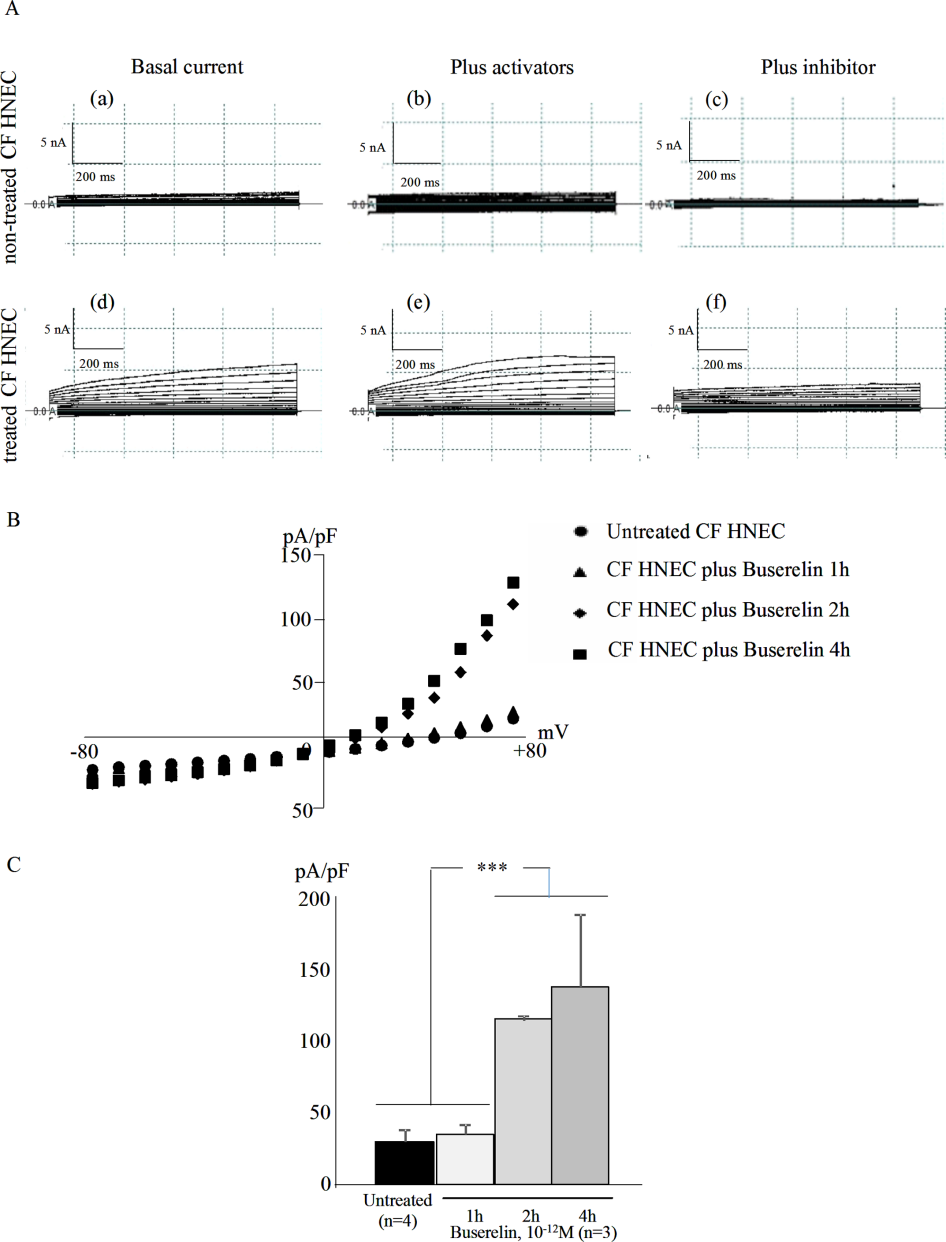
Patch-clamp analysis of the chloride channel function of CFTR in CF HNEC in the presence of buserelin. **A.** Representative current traces were recorded in non-treated CF HNEC at the basal level (a), in the presence of CFTR activators (Fsk/Gst) (b), in the presence of CFTR inhibitor (CFTR_inh_172) (c). Current traces were recorded in buserelin treated CF HNEC at the basal level (d), in the presence of CFTR activators (Fsk/Gst) (e), in the presence of CFTR inhibitor (CFTR_inh_172) (f). **B.** I/V curves with normalized currents by cell capacitance (pA/pF) are presented for CFTR basal current in CF HNEC, with and without buserelin treatment (1, 2 or 4 hours). **C.** Mean CFTR-related normalized current amplitudes were recorded at +80 mV and are presented as bar graphs. The statistical analysis indicated that CFTR-related currents were highly increased in the presence of buserelin (2 and 4 hours). Data are presented as mean ± S.E.M. for n = 4. Student’s t test was used to evaluate significant differences (***: p<0.001).

We showed that GnRH-R is expressed in ALI cultures of HNEC. The next step of our study was to assess the effect of buserelin upon the Cl^-^ channel function of F508del-CFTR. To measure the function of CFTR in CF HNEC, automatic patch-clamp was used in the whole-cell configuration [16, 32]. Representative current traces obtained in non-treated and buserelin treated CF HNEC are shown in Fig 3A. We showed that the amplitudes of the currents were higher in the presence of buserelin and that buserelin permits CFTR to respond to its specific activators and inhibitor. Higher levels of currents were observed after buserelin treatment, as showed by I/V curves (Fig 3B). These curves for CFTR current were obtained from CF HNEC without and in the presence of buserelin (1, 2 or 4 hours) and they indicated that buserelin has a positive effect upon the Cl^-^ channel function of F508del-CFTR. The amplitude of the CFTR-related currents recorded at +80mV were used for the statistical analysis and are presented as bar graph in Fig 3C. We found that CFTR currents were significantly increased in the presence of buserelin in CF HNEC. This result is fully in accordance with previous work using purified GnRH in CFBE41o2/F508del cells [31]. Nevertheless, because whole cell configuration was used, it is not possible to assert that buserelin acts upon the intrinsic function of F508del-CFTR. The increased Cl^-^ effluxes could also be due to an increased maintenance of some F508del-CFTR in membranes, to a decreased recycling or to an increased membrane targeting. According to our previous results, an increased presence within membranes is probably seen with buserelin [15, 31]. Indeed, we previously showed this effect with purified GnRH [31].

**Fig 4:**
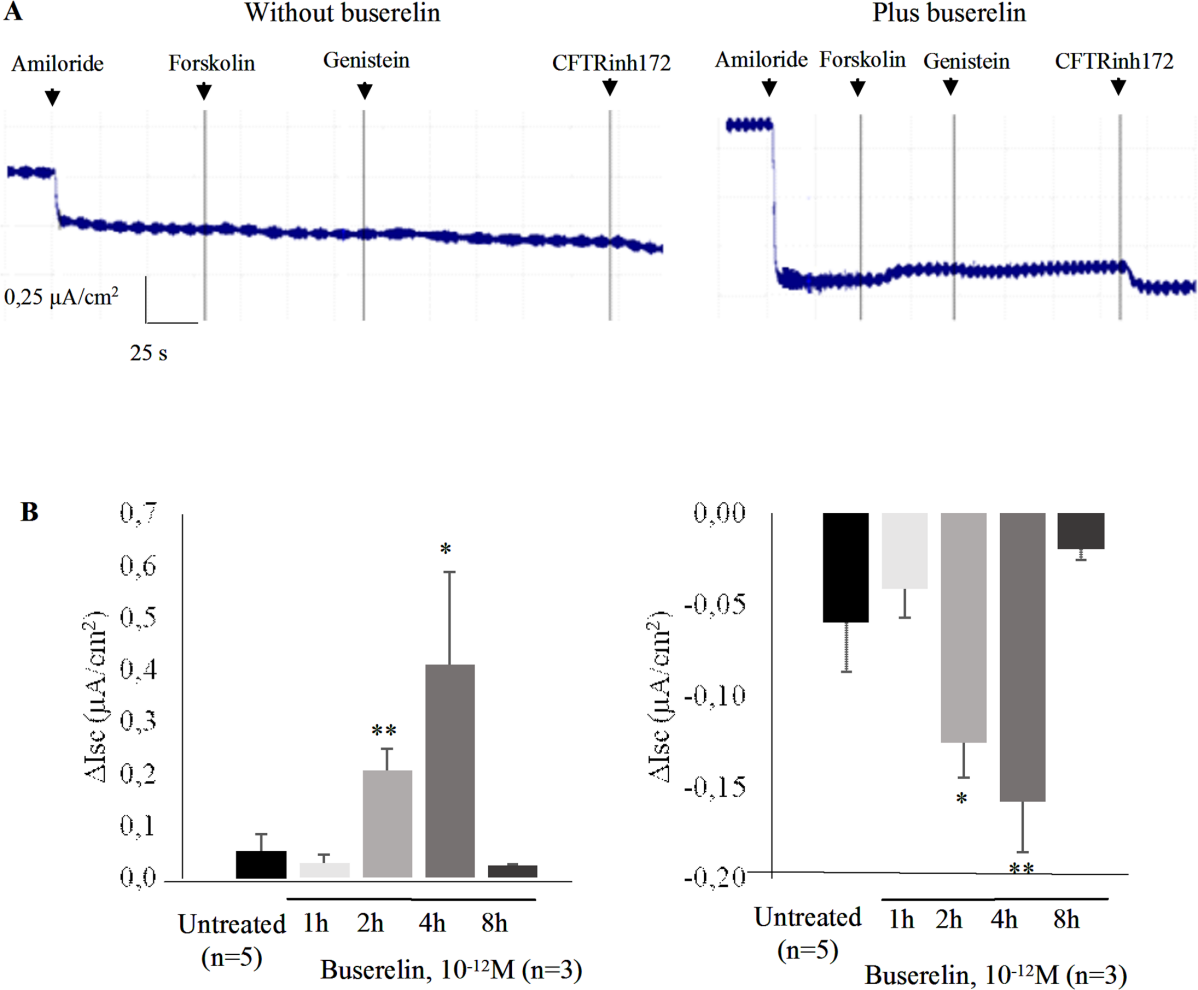
Measurement of transepithelial currents in CF HNEC by Ussing Chamber. **A.** Example of representative traces of short-circuit currents (Isc) in air-liquid culture in CF HNEC, recorded in the absence (left panel) and in the presence (right panel) of buserelin (2h, 10^-12^M). Amiloride (20 μM), forskolin (Fsk, 10 μM), genistein (Gst, 50 μM) and the CFTRinh172 (10 μM) were added sequentially. **B.** Bar graph representation for transepithelial Cl^-^ conductance (ΔIsc μA/cm^2^) in CF HNEC at air-liquid culture in low Cl^-^ Krebs bath, after buserelin treatment (10^-12^M; 1, 2, 4 and 8h) with Fsk/Gst (left panel) and CFTRinh172 (right panel). Whereas transepithelial Cl^-^ conductance was increased at the 2 and 4h time points, it was aboslished at 8h. Data are presented as mean ± S.E.M. (n = 4). Student’s t test was used to evaluate significant differences (*: p<0.05, **: p<0.01).

Transepithelial currents in CF HNEC were measured by Ussing Chamber. Traces of short-circuit currents (Isc) in ALI cultured CF HNEC, without (Fig 4A, left panel) and in the presence of buserelin (Fig 4A, right panel) were recorded. It was noticed that CFTR’s activators and inhibitor were more efficient in treated cells. A Kristal-Wallis test of the currents was performed to assess the statistical difference between various time points (1, 2, 4 and 8 hours). Distribution is presented as bar graphs in Fig 4B. The bar graphs of transepithelial Cl^-^ conductance in CF HNEC in low Cl^-^ Krebs bath after buserelin treatment with Fsk/Gst and CFTRinh172 (Fig 4B) show significant increased transepithelial currents in treated CF HNEC at the 2 and 4 h time points. Nevertheless, the effect of buserelin is likely transient since at the 8 h time point, currents were deeply decreased. This observation can be related to previous results obtained by label-free whole cell assays using a biosensor to detect a ligand-induced cellular response by acoustic and electrical signals [40]. It was described and measured that the persisted signaling effect of GnRH was due to its rebinding while the signaling effect of buserelin is a combination of both rebinding and prolonged drug-target occupancy [41]. The binding kinetic of a drug-target interaction, rather than its equilibrium binding parameters is important for *in vivo* efficacy. It is accepted that buserelin presents a slow dissociation rate from the receptor [42], GnRH and buserelin K_D_ being 2.9 and 0.04nM, respectively [43]. Therefore, its rebinding is low, affecting its long term effect. Furthermore, the membrane degradation of buserelin has to be taken into account since it was shown to be degradated by rat kidney membrane fraction [44]. Therefore, membrane degradation by airway epithelial cells cannot be excluded; despite it has never been studied to our knowledge.

**Fig 5:**
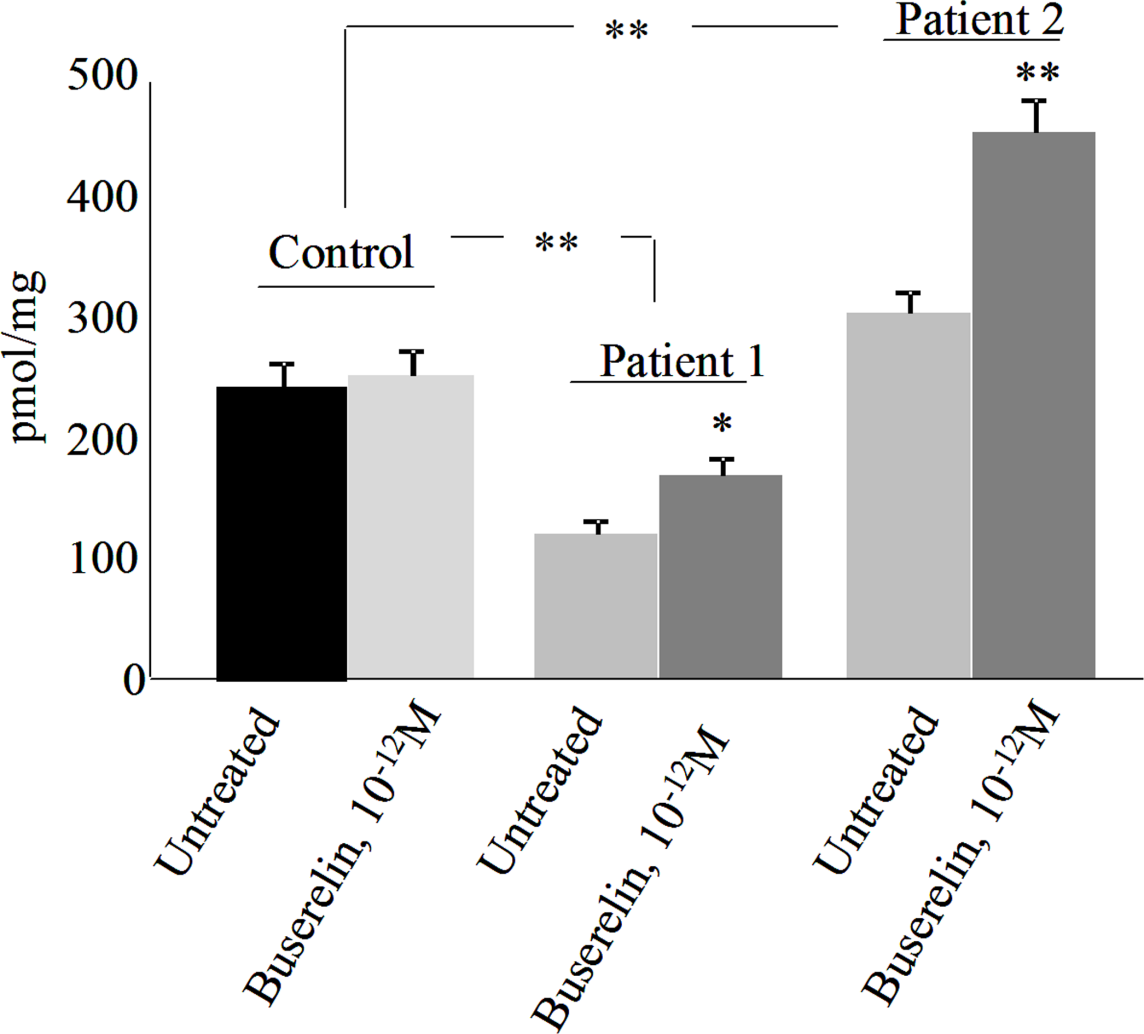
cAMP measurement in control and CF HNEC, with or without Buserelin treatment (10^−12^ M, 2h). cAMP was assessed in control (n = 2, in triplicate) and in CF HNEC (n = 2, in triplicate). Results are expressed in pmol cAMP/mg of total proteins. Beside an observed variability between patients, cAMP was increased in CF cells after buserelin treatment. Data are mean ± S.E.M. Student’s t test was used to evaluate the significance (*: p<0.05).

The GnRH-R signaling network is complex (for review, [45]). GnRH-R activates G-proteins, leading to the activation of adenylyl cyclase and phospholipase C (PLC). Adenylyl cyclase then generates cAMP, stimulating protein kinases A (PKA) whereas PLC activates protein kinases C (PKC). It also elevates the cytoplasmic Ca^2+^ concentration which activates calmodulin. Because CFTR is activated by PKA and PKC [46], because it was very recently shown that CFTR activation by calmodulin is due to an interaction between the regulatory region of CFTR and calmodulin [47], and because CFTR is a cAMP-dependent channel [47], it’s activation by GnRH is likely obvious. Because cAMP is increased by GnRH, we assessed its level after buserelin treatment. cAMP measurement was performed in control and CF HNEC, with or without Buserelin treatment (Fig 5). cAMP was found to be increased in cells from CF patients after buserelin treatment, whereas in control it was not modified. Because GnRH increases cAMP, it can be speculated that control cells and CF cells respond differently to GnRH. This latest point needs further investigations. Despite a great variability regarding basal levels of cAMP between patients, the effect of buserelin was the same in both of them. Therefore, we suggest that the benefic effect of buserelin upon F508del-CFTR Cl^-^ channel function is due, at least in part, to an elevation of cAMP within cells. In a previous work using another cell type, we showed that a treatment by GnRH induces an increased amount of F508del-CFTR within cell membranes [31]. Therefore, both effects are likely involved in buserelin treated cells.

**Fig 6:**
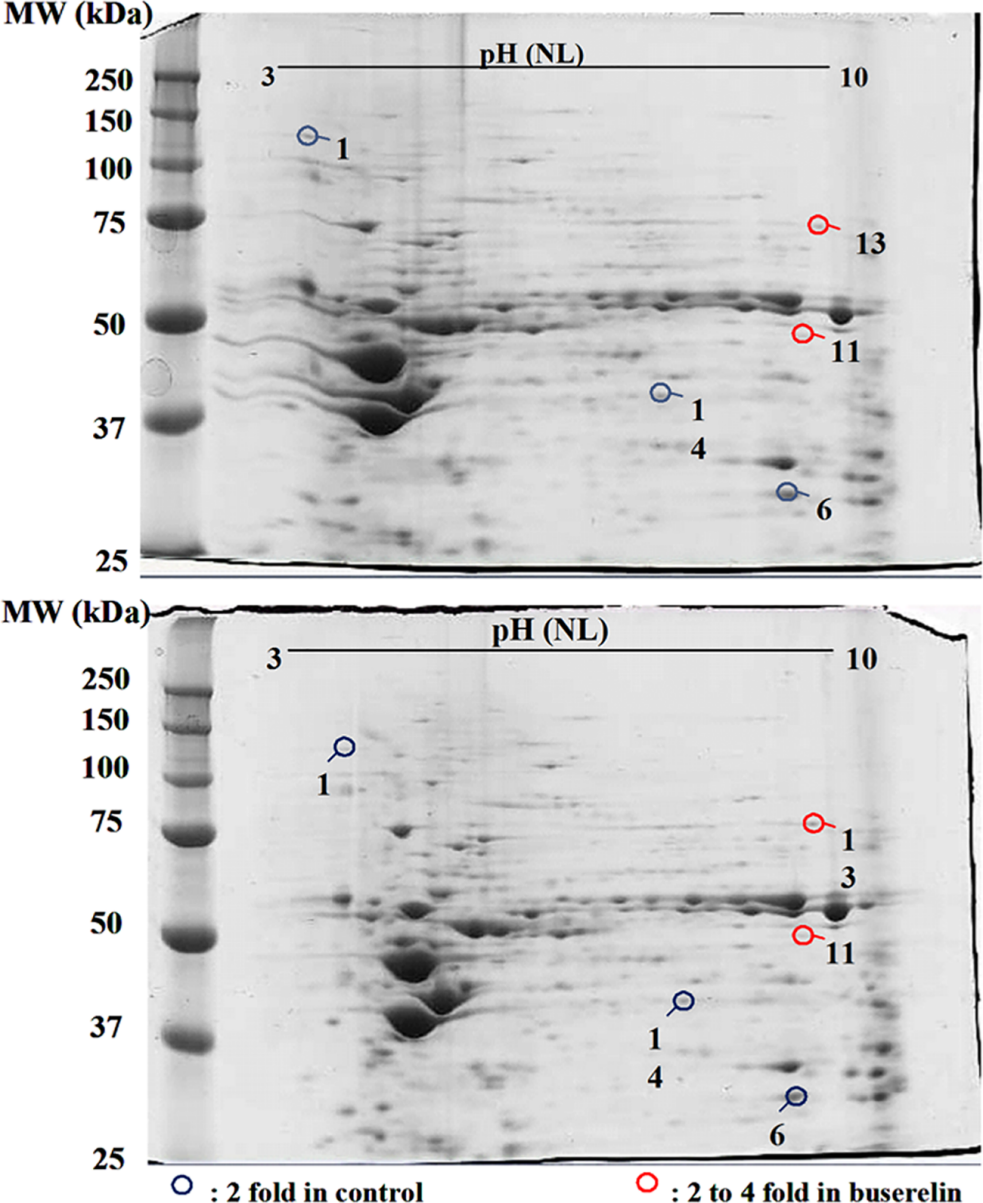
Identification of differentially expressed proteins in CF HNEC after buserelin treatment (10^-12^M, 2h). Two-dimensional gel analysis of differentially expressed proteins in untreated (upper gel) and in treated CF HNEC (lower gel) was performed. 14 spots with increased or decreased intensities (PDQuest analyzis) were submitted to MS. 7 Spots in blue and red circles were down-regulated and up-regulated, respectively, after treatment and were further analyzed regarding their possible involvement in CF (Table 1).

In order to search for further pathways involved in the positive effect of buserelin upon F508del-CFTR’s function, we performed 2-DE with the aim of looking for differentially expressed proteins, before and after treatment of the cells. Examples of 2-DE gels are shown in Fig 6. 14 spots with increased or decreased intensities in the presence of buserelin were submitted to MS. Among them, 7 down- and up-regulated spots after treatment were further analyzed regarding their possible involvement in CF. 6 proteins were identified: Serpin B3, NADH dehydrogenase (NDUV1), Voltage-dependent anion-selective channel protein 2 (VDAC2), Endoplasmin, ATP synthase subunit alpha (ATPA) and T-complex protein 1 subunit zeta (TCPZ) (Table 1).

**Table 1:** Identified proteins by MS.

Spot Number	UniProt Access. Number	Protein Name	Gene	Sequence coverage	kDa/Pi	Number of Spectra	Unique	Expression (after Buserelin treatment)
**14**	P29508	SPB3_HUMAN (Serpin B3)	SERPIN B3	60	44.4/6.35	64	28	decreased
**11**	P49821	NDUV1_HUMAN (NADH dehydrogenase [ubiquinone] flavoprotein 1, mitochondrial)	NDUFV1	10	49.7/8.51	6	5	increased
**6**	P45880	VDAC2_HUMAN (Voltage-dependent anion-selective channel protein 2)	VDAC2	55	31.5/7.5	109	31	decreased
**1**	P14625	ENPL_HUMAN (Endoplasmin)	HSP90B1	5	92.3/4.76	4	4	decreased
**12**	P25705	ATPA_HUMAN (ATP synthase subunit alpha, mitochondrial)	ATP5A1	13	59.6/9.16	6	6	increased
**13**	P40227	TCPZ_HUMAN (T-complex protein 1 subunit zeta)	CCT6A	25	57.9/6.24	14	14	increased

Serpins are serine and cysteine proteases inhibitors involved in proteolysis in inflammation and protein folding [48]. Beside their anti-protease activity, they also act as chaperones or hormone transporters [49]. Serpin B3 (previously known as SCCA1) is mainly localized in the cytosol. It displays an anti-apoptotic function and is associated with drug resistance suggesting that it favors tumor cell survival, despite a poorly understood molecular mechanisms (for review: [50, 51]). In the lung, Serpin B3 overexpression has been correlated to pulmonary epithelial dysfunction of pulmonary fibrosis [52, 53]. Its decreased expression after buserelin treatment is likely in favor of a cell protection.

Mitochondria play a principal role in oxidative metabolism in which nutrients are converted into energy. The electron transport chain is composed of five multimeric protein complexes (Complex I–V). Complex I (also called mitochondrial NADH dehydrogenase complex (ubiquinone)) is the main entry point for electrons. It is positively modulated by the activity of CFTR and negatively regulated when the expression and/or the function of CFTR is altered [54]. Therefore, because we found that its expression is increased after buserelin treatment, we can correlate this finding with the restoration of F508del-CFTR function by the treatment. Our finding that its increased expression is due to buserelin is reinforced by previous results showing that there is a decrease in Complex I activity in CFBE41o- cells, when compared to 16HBE14o- cells [54].

Voltage Dependent Anion-selective Channels (VDACs) are expressed in almost all mammalian tissues and are the most abundant proteins of the outer membrane of mitochondria. These channels present a wide pore used by mitochondria to exchange solutes such as ADP/ATP, NADH with the cytosol [55]. Among VDACs, VDAC2 has a distinctive role in mediating sarcoplasmic reticulum to mitochondria local Ca2+ transport and is involved in apoptosis [55]. Nevertheless, how VDAC2 rules various functions such as channeling, recruiting and promotion/inhibition of other proteins is unknown [55]. Studies of the role of mitochondria in CF have demonstrated that there is a reduction in mitochondrial Complex I electron transport chain function due to an alteration of the pH of Complex I, a reduced expression of the mitochondrial gene encoding a Complex I subunit and to oxidative modifications of Complex I subunits [56]. Specific mitochondrial impairments associated with the F508del mutation were observed, such as oxygen consumption, adenine nucleotide translocator-dependent ADP/ATP exchange and both mitochondrial Complex I and IV activities [57]. The fact that VDAC2 expression is decreased after buserelin treatment in our cells is likely due to the F508del mutation itself. In our opinion, the decreased expression of VDAC2 does not explain the restoration of the function of F508del-CFTR observed here.HSP90 proteins are highly conserved molecular chaperones that have key roles in signal transduction, protein folding, protein degradation, and morphologic evolution. HSP90 proteins normally associate with other cochaperones and play important roles in folding newly synthesized proteins or stabilizing and refolding denatured proteins after stress. HSP90B1 is an endoplasmic reticulum HSP90 protein. HSP90 proteins are highly conserved molecular chaperones that have key roles in signal transduction, protein folding, protein degradation, and morphologic evolution. HSP90 proteins normally associate with other cochaperones and play important roles in folding newly synthesized proteins or stabilizing and refolding denatured proteins after stress. HSP90B1 is an endoplasmic reticulum HSP90 protein. Other HSP90 proteins are found in cytosol (see HSP90AA1; 140571) and mitochondria

Heat Shock Proteins 90 (HSP90) proteins are conserved molecular chaperones with a key role in protein folding and degradation [58]. They associate with other co-chaperones and play important roles in folding newly synthesized proteins or stabilizing and refolding denatured proteins after stress. Endoplasmin (HSP90B1) is an ER HSP90 protein. CFTR protein interacts with a large variety of molecular chaperones, including HSP90B1, involved in its folding, disassembly, and translocation, [58, 59]. Furthermore, endoplasmin is expressed in HNEC where it is deregulated when CFTR gene is mutated [60]. Because we found that endoplasmin is decreased after buserelin treatment, it is likely involved in the increased function of F508del-CFTR which was observed here. Indeed, changes in the interactome, including a decreased expression of HSP90B1, were coupled to the appearance of fully glycosylated from of F508-CFTR (Band C, [61]).

ATP synthase subunit alpha, mitochondrial (ATP5A1) is a mitochondrial membrane ATP synthase (Complex V). Its role is to produce ATP from ADP in the presence of a proton gradient across the membrane which is due to electron transport complexes of the respiratory chain. Previous results showed that the function of ATP5A1 is not altered in cells expressing F5087del-CFTR [56, 57]. In our experiments, we found an increased expression of ATP5A1. Because it produces ATP which is necessary to the Cl^-^ channel function of CFTR, it is probably involved in the positive effect of buserelin treatment.

Chaperonin containing T-complex polypeptide 1 (CCT6A) is the zeta subunit of the chaperonin containing TCP1 complex (CCT). This complex is composed of two similar rings, each containing eight different proteins (alpha, beta, gamma, delta, epsilon, eta, theta and zeta; for review: [62]). Each subunit of the ring has three domains: an ATP binding site, an unfolded protein binding domain and a region which connects the two other regions. Unfolded proteins enter the complex and are folded in an ATP-dependent manner, and it is estimated that up to 15% of all cellular proteins interact with CCT [63]. CCT6A is a chaperon for cytoskeletal proteins such as actin and tubulin [64] but it also assists the folding of other proteins. Interestingly, CCT 6A expression was reported to be regulated by a sexual hormone (progesterone), at least in hens. Therefore, its increased expression is likely due to buserelin and may first favor F508del-CFTR folding and second favor its membrane localization and function due to its action on actin. We thus depicted differentially expressed proteins before and after buserelin treatment. These proteins are potentially involved in the restoration of some Cl^-^ channel function of F508del-CFTR, after treatment. Nevertheless, further experiments will unsure their specific role in the restoration process of F508del-CFTR function.

**Fig 7:**
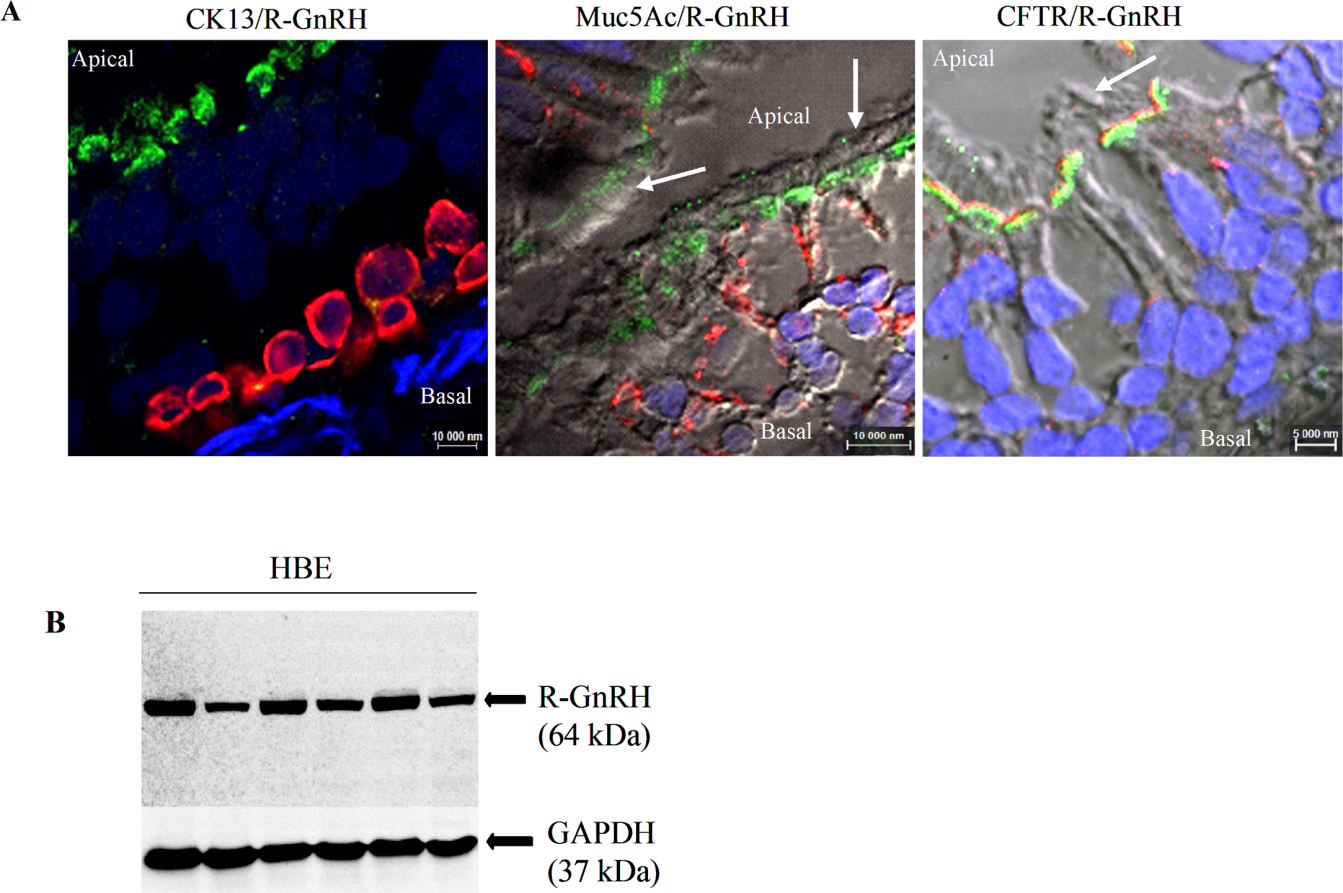
Localization and expression of GnRH-R in HBEC. **A.** Representative confocal images of HBEC co-stained by cell markers CK13 (mucous cells, left panel, red labelling) and Muc5AC (ciliated cells, middle panel, red labelling) and GnRH-R (green labelling). Co-distribution of GnRH-R (green labelling) and CFTR (red labelling) was observed in ciliated cells, in apical cell membranes. **B.** Representative immunoblot performed to assess GnRH-R expression in 6 different HBEC cultures.

Because we found that buserelin alleviates Cl^-^ channel defect in HNEC and because HBEC are the target of most pharmaceutical compounds in CF, we studied GnRH-R localization and expression in human bronchus. As shown by the representative confocal images obtained by confocal microscopy (Fig 7A), GnRH-R is present in ciliated cells and is co-distributed with CFTR with an apical localization. Immunoblots performed with proteins from HBEC (Fig 7B) indicated that the protein is present in cultured cells.

## Conclusion

In conclusion, we show here that the GnRH-R is expressed in normal and CF HNEC, despite its exact physiological role in airways is unclear. We also show that the GnRH analog buserelin restores some F508cel-CFTR’s Cl- channel function in CF HNEC. This alleviation of the Cl^-^ transport defect in F508del-CFTR cells is accompanied by a modulation of the expression of proteins such as Serpin B3, NDUV1, ATP5A1 and TCPZ which likely explain the effect of buserelin. Because buserelin is already used in medical practice as nasal sprays, we propose that it is a candidate that has to be tested in human, knowing that we show here that GnRH-R is also expressed in human bronchus. Nevertheless, while buserelin is already used in the clinic, many side effects have been reported in various cell and animal models, likely due to internalization and downregulation of type I mammalian GnRH-R [65]. It may induce severe gut dysmotility and enteric neurodegeneration in rat [66], apoptotic cell death and decreased diameter and epithelium thickness of seminiferous tubules in the adult rat testes [67]. In human, after a 6- to 9-month period of treatment by buserelin, side effects were observed [68]. They are reported to be hot flushes, dyspareunia and decreased libido. Hemogram, urine analysis, serum biochemical and hormonal tests are reported to remain in the normal range. Furthermore, the ovulatory cycle is reported to be rapidly returned to normal when the treatment is stopped. In order to avoid side effects and to target lungs, aerosolization for short periods of time should be used.

## Abbreviations

GnRH: Gonadotropin-Releasing Hormone
CF: Cystic Fibrosis
CFTR: Cystic Fibrosis Transmembrane Regulator
